# Crystalline lens dislocation as a presenting sign of Streptococcus pyogenes invasive infections

**DOI:** 10.1099/acmi.0.000903.v4

**Published:** 2025-05-27

**Authors:** Paulina Liberman, Jacob G. Light, Shazia Dharssi, Tracy Howard, Jennifer Lu, Patricia J. Simner, Karen C. Carroll, Cheng-Ying Ho

**Affiliations:** 1 Ocular Immunology Division, Wilmer Eye Institute, Johns Hopkins University School of Medicine, Baltimore, USA; 2 Patient Access Center for the Eye, Wilmer Eye Institute, Johns Hopkins University School of Medicine, Baltimore, USA; 3 Oculoplastics Division, Wilmer Eye Institute, Johns Hopkins University School of Medicine, Baltimore, USA; 4 Microbiology Laboratory, Johns Hopkins Hospital, Baltimore, USA; 5 Microbiology Division, Department of Pathology, Johns Hopkins University School of Medicine, Baltimore, USA; 6 Division of Neuropathology and Ophthalmic Pathology, Department of Pathology, Johns Hopkins Hospital, Baltimore, USA

**Keywords:** intravitreal injections, invasive streptococcal infections, lens dislocation, ocular infection, *Streptococcus pyogenes*, toxic shock syndrome

## Abstract

**Introduction.** To describe two cases of crystalline lens dislocation as a presenting feature of invasive group A *Streptococcus* (GAS) infection and its management.

**Case presentation.** We report on a 58-year-old woman and a 36-year-old man who presented in 2024 with acute vision loss and severe ocular and systemic symptoms. Both patients were found to have lens dislocation and were diagnosed with invasive GAS infection. The 58-year-old woman had a complicated clinical course leading to enucleation, while the 36-year-old man responded favourably to early and aggressive treatment with systemic and intravitreal antibiotics. The responsible GAS strains were sequence type (ST) 28 and ST433, respectively.

**Conclusion.** These cases highlight the importance of recognizing crystalline lens dislocation as a potential sign of ocular GAS infection. Two specific strain types of GAS associated with these findings, ST28 and ST433, are reported. In patients with GAS sepsis presenting with corneal oedema and zonular loss, clinicians should immediately initiate treatment, including intravitreal antibiotic injections and systemic therapy. Prompt and aggressive management can be crucial in preserving ocular structures.

## Data Summary

All data associated with this work are reported within the article and supplementary file. Sequences were deposited in National Center for Biotechnology Information under the accession numbers: SRX27746061 and SRX27746060.

## Introduction

Lens dislocation, although rare, is a severe complication that can arise from ocular infections. *Streptococcus pyogenes* [group A *Streptococcus* (GAS)] is a known pathogen capable of causing invasive infections (iGAS) with significant ocular morbidity [1]. A range of ocular diseases can be associated with GAS, including conjunctivitis, keratitis, endophthalmitis and poststreptococcal uveitis. Endophthalmitis due to GAS is rare but has a poor prognosis, requiring aggressive treatment and sometimes resulting in enucleation. Poststreptococcal uveitis, an immune-mediated response to GAS infection, typically presents as bilateral nongranulomatous anterior uveitis and responds to corticosteroids [2,4].

Alerts from health organizations worldwide emphasize the growing concern over iGAS. In 2022 and 2023, multiple countries, including France, Ireland, the Netherlands, Sweden, the United Kingdom and the United States, reported a significant increase in cases of iGAS [5,8]. In the United States, streptococcal toxic shock syndrome is a notifiable disease [9]. A recent increase in cases has coincided with the emergence of the hypervirulent M1UK lineage, whose prevalence rose from 1.7% in 2015–2018 to 11% in 2019–2021 [10].

Herein, we report two cases, diagnosed in 2024, in which crystalline lens dislocation was the presenting feature of GAS-induced toxic shock syndrome.

## Case reports

### Case 1

**Fig. 1.**
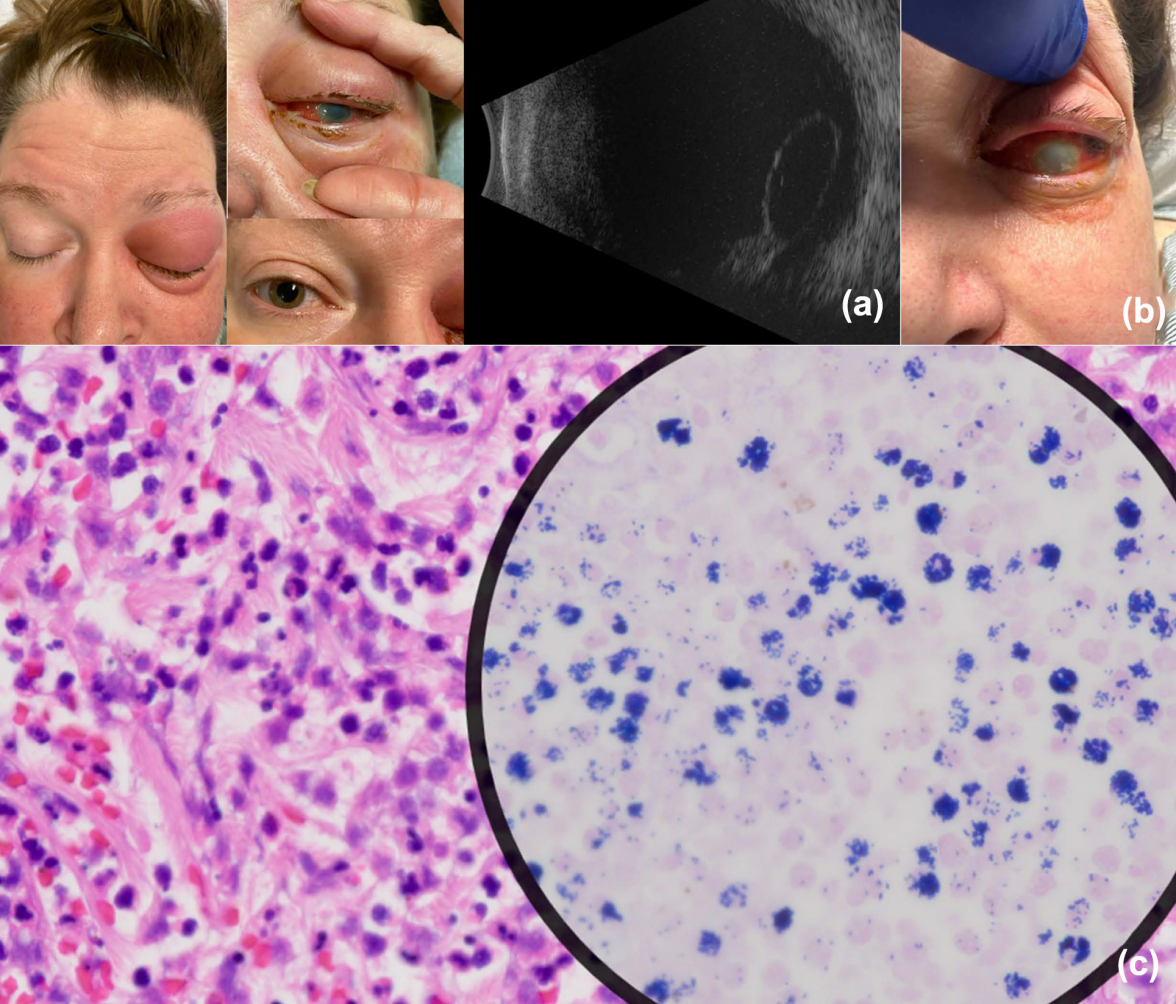
Clinical findings and pathology of a 58-year-old woman with invasive GAS ST28 infection and lens dislocation. (a) An external photograph showing lid erythema, conjunctival injection and left corneal opacity, an ultrasound reveals dislocation of the crystalline lens into the vitreous cavity. (b) External photograph taken two days after admission shows conjunctival injection, severe corneal opacity and scleral perforation. (c) Pathology of the enucleated eye demonstrates an overview of the abscess filling the entire chamber and a Gram stain shows gram-positive cocci.

A 58-year-old woman presented with a one-day history of vision loss and ocular pain in the left eye. Her past medical history included treated cutaneous melanoma of the leg, excised eleven years prior without recurrence. At the time of presentation, the visual acuity of her left eye was counting fingers and the intraocular pressure (IOP) was markedly elevated at 50 mm Hg. A slit lamp examination revealed conjunctival injection and corneal oedema. She was initially suspected of having acute-angle-closure glaucoma and was treated with a laser peripheral iridotomy and IOP-lowering medications including mannitol, acetazolamide, timolol, brimonidine and dorzolamide. She was evaluated the following day at the Wilmer Eye Institute. Ophthalmologic examination revealed an opaque cornea, conjunctival redness and discharge. The visualization of intraocular structures was severely limited by the corneal opacity. Ocular B-scan ultrasonography revealed an ovoid, well-circumscribed, hyperreflective opacity with low internal reflectivity noted in the vitreous cavity, resting inferiorly, consistent with a fully dislocated crystalline lens. The vitreous was otherwise echolucent (Fig. 1). The patient appeared acutely ill on systemic examination and was referred to the emergency department (ED). On admission, the patient was febrile (103.1 °F), tachycardic and hypotensive. A laboratory evaluation showed a white blood cell count of 11,140 cells µl^−1^ (84% neutrophils), lactic acid of 4.1 mmol l^−1^ and elevated liver enzymes, consistent with septic shock. She required low-dose vasopressors and responded to volume resuscitation but developed severe diarrhoea with electrolyte imbalance. A conjunctival sample obtained on a swab inoculated to 5% sheep blood agar (SBA) and chocolate agar grew heavy GAS, leading to the initiation of systemic ceftriaxone and linezolid (Fig. S1a, available in the online Supplementary Material). Antimicrobial susceptibility testing (AST) was not performed. Computed tomography imaging revealed preseptal cellulitis and confirmed left lens dislocation into the vitreous humour. The left cornea developed complete opacity, and 48 h after admission, the sclera perforated, necessitating enucleation. No orbital involvement was noted intraoperatively. Histopathologic examination revealed an abscess in the anterior chamber, with Gram Weigert staining notable for linear clusters of Gram-positive cocci (Fig. 1). Whole-genome sequencing (WGS) [6] was performed using short-read Illumina sequencing (2×300 P2 reagents) as previously described on the NextSeq1000 (Illumina, San Diego, CA) and deposited to the sequence read archive under bioproject PRJNA1226217. An analysis was performed using the PHoeNIx pipeline (https://github.com/CDCgov/phoenix/wiki) [11]. WGS revealed the strain corresponded to sequence type 28 (ST28). No antimicrobial resistance genes nor virulence factor genes were identified.

### Case 2

A 36-year-old previously healthy man, with recent travel to Hawaii and an adult contact with impetigo, presented to the ED complaining of recent onset acute vision loss in the left eye, and systemic symptoms including chills, exanthema, myalgias, nausea, diarrhoea and diffuse abdominal pain. Examination in the ED revealed sacral impetigo. A laboratory evaluation revealed a white blood cell count of 19,940 cells µl^−1^ (47% band neutrophils and 43% segmented neutrophils), lactic acid of 6.1 mmol l^−1^ and elevated liver enzymes and creatinine, consistent with septic shock. Skin swabs inoculated to 5% SBA and two sets of blood cultures obtained in BACTEC aerobic resin and anaerobic bottles (BD Diagnostics, Inc. Sparks, MD) confirmed GAS infection. Immediate treatment included fluid resuscitation, intravenous immunoglobulin (IVIG) and broad-spectrum antibiotics (cefepime, vancomycin, doxycycline and metronidazole) (Fig. S1b) . After confirmation of GAS infection, the initial broad-spectrum regimen was narrowed to ceftriaxone and clindamycin.

**Fig. 2.**
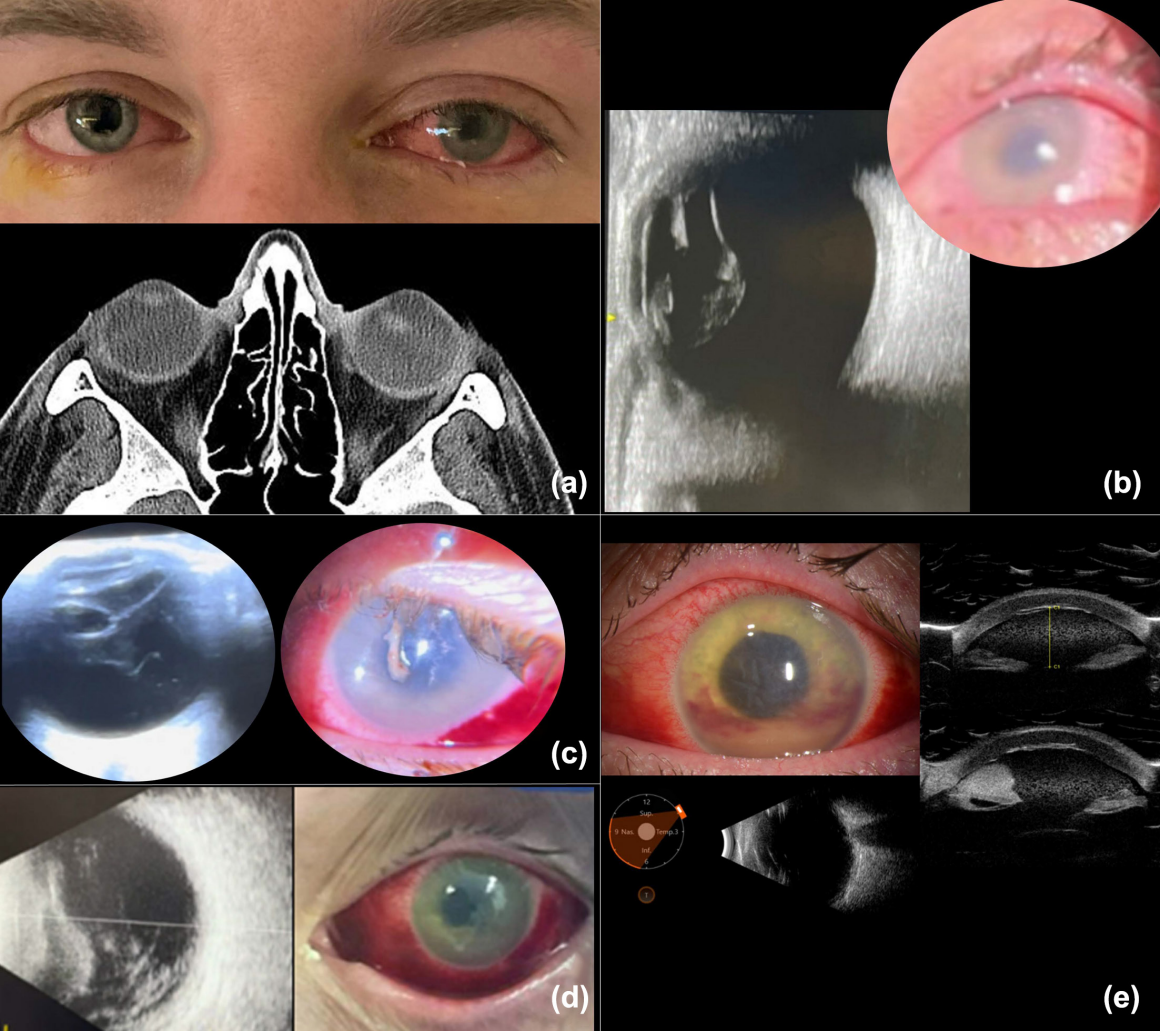
Initial findings and clinical course of a 36-year-old man with invasive GAS ST433 infection and lens subluxation (*quality of b, c and d limited due to the patient’s critical condition and images taken at bedside*). (a) An external photograph of the eyes at presentation shows conjunctival injection of the left eye, corneal oedema and dyscoria. A computed tomography scan reveals the abnormal position of the left lens. (b) An external photograph 40 h after symptom onset demonstrating increased corneal oedema. An ultrasound confirms the subluxation of the crystalline lens and no vitreous opacities. (c) An external photograph taken on the first day after injection of intravitreal antibiotics, showing corneal oedema and development of hypopyon. An ultrasound shows the development of vitreous opacities. (d) An external photograph on the third day after the first injection showing subconjunctival haemorrhage, persistent corneal oedema and hypopyon. An ultrasound suggests worsening of the vitreous opacities. (e) An external photograph showing corneal opacity and hypopyon with layering hyphaema. An ultrasound indicates the presence of hypopyon, with no clear view of the crystalline lens on examination or ultrasound.

**Fig. 3.**
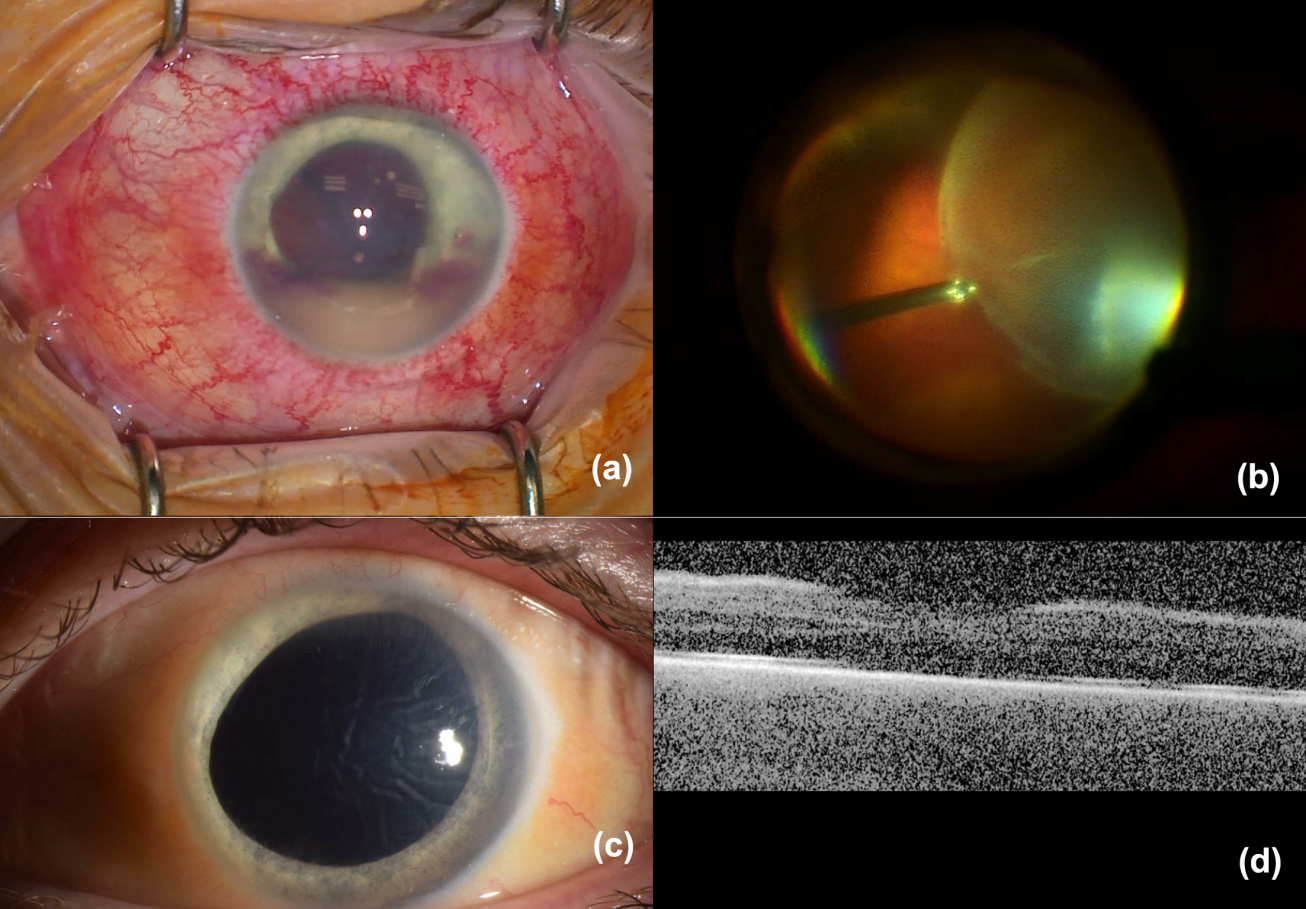
Intraoperative findings and postoperative results of a 36-year-old man with invasive GAS ST433 infection and lens subluxation. (a) An intraoperative photograph depicting diffuse injection, corneal oedema and hypopyon. (b) An intraoperative photograph showing an intact, albeit nasally dislocated crystalline lens that underwent lensectomy. (c) A postoperative external photograph showing no injection, persistence of corneal folds, resolution of the hypopyon and aphakia. (d) A postoperative optic coherence tomography of the retina shows preservation of the foveal contour (*quality limited by corneal folds and oedema*).

The initial ED examination noted bilateral conjunctival injection and dyscoria of the left eye (Fig. 2). Magnetic resonance imaging of the brain showed subluxation of the crystalline lens. Ophthalmologic evaluation within 12 h of admission revealed visual acuity of J1 in the right eye and hand movement in the left eye. The examination of the right eye was normal except for mild conjunctival injection. The left eye had an IOP of 25 mm Hg and developed significant corneal oedema within hours of admission, precluding fundus visualization. B-scan ultrasound showed a subluxated lens without vitreitis (Fig. 2). The eye received an intravitreal injection of ceftazidime and clindamycin. Topically, prednisolone acetate and atropine were commenced. The following day, the left eye developed hypopyon and vitreous opacities. AST of the isolates performed on the BD Phoenix Automated instrument using SM101 cartridges (BD Diagnostics, Inc.) showed clindamycin resistance; systemic clindamycin was switched to linezolid. The patient received IVIG for 3 days. On the third day, intravitreal injections of ceftazidime, vancomycin and dexamethasone were administered. By the fourth day, the systemic infection was deemed sufficiently controlled to allow the addition of oral prednisone. Ten days postadmission, persistent corneal folds, hypopyon and poor visualization of the crystalline lens on clinical exam and echography raised concerns about potential lens disruption exacerbating intraocular inflammation. A decision was made to repeat ceftazidime and dexamethasone injections, and anterior chamber washout, vitrectomy and lensectomy were planned. Surgery was performed 2 weeks after admission, revealing a nasally subluxated crystalline lens with temporal zonular dehiscence but an intact lens capsule. Pars plana lensectomy and vitrectomy were performed (Fig. 3). Aspirates from the anterior chamber and vitreous cavity were analysed. The cultures of the aspirates were negative, and histopathology and Gram staining did not show the presence of cocci. The patient had a favourable clinical course, and prednisone was tapered. Thirty days postsurgery, the patient had persistent corneal oedema, his anterior chamber and vitreous were clear and his vision corrected to 20/100 with a +12 D lens (Fig. 3). Sequencing performed as in Case 1 of GAS isolated from the positive blood culture showed the strain corresponded to ST433. Consistent with phenotypic AST, the *ermA* (erythromycin ribosome methylase) gene associated with clindamycin resistance was detected, among other resistance genes (*aph(3’)-IIIa* (aminoglycoside phosphotransferase gene), *sat4* (streptothricin acetyltransferase gene) and *tetM* (tetracycline ribosomal protection enzyme). No virulence factor genes were identified.

## Discussion

We have described two cases of invasive GAS with severe ocular involvement. Interestingly, the chief complaint of both patients was acute vision loss, resulting from fulminant corneal oedema and atraumatic crystalline lens subluxation. This is an unusual presentation of invasive GAS disease.

Sangave *et al*. [1] described a similar presentation in a 51-year-old healthy man presented with eye pain and blurred vision. Of note, like both of our cases, this patient also had corneal oedema, Descemet’s folds and ocular hypertension. At the time of ophthalmological examination 30 h after presentation to the ED, he was noted to have a posterior dislocation of the crystalline lens. He was injected with vancomycin, ceftazidime and dexamethasone. Unfortunately, his vision declined to no light perception at 3 months of follow-up with subsequent phthisis [1].

In case 1, in which presentation was delayed more than a day after the symptoms had started, the patient’s clinical course was dire, with the development of fulminant infectious scleritis, perforation and loss of the affected eye. On the other hand, the patient in case 2 presented rapidly after symptom onset. This led to early systemic treatment with broad-spectrum antibiotics, initiated just 2 h after symptom onset, and IVIG. Intravitreal antibiotics were injected 41 h after symptom onset. We propose that the very early initiation of aggressive systemic and ocular management may have been crucial in the preservation of the eye structure and visual potential in this case. Interestingly, at this very early time point, the patient had corneal opacity and lens subluxation but no hypopyon or vitreous opacities on ultrasound to suggest manifest endophthalmitis (Fig. 3). Injecting promptly even in the absence of evident vitreous involvement was paramount in this case. In terms of the medications injected, initially, we chose clindamycin and ceftazidime because the patient had a positive blood culture for GAS. In the treatment of systemic invasive GAS, clindamycin is added to penicillin or cephalosporin because it decreases the expression and production of group A streptococcal virulence factors and exotoxins [12]. We extrapolated this rationale for the treatment of eye involvement. On later injections, once the AST had been performed, the antibiotic therapy was adjusted according to the susceptibility profile. Also, dexamethasone was added in the later injections once a more prominent inflammatory response was shown in the vitreous and anterior chamber. The use of IVIG was indicated for the treatment of systemic disease. IVIG can neutralize streptococcal virulence factors and modulate the immune response [13]. The impact of IVIG on the eye prognosis remains unknown.

Classically, strains of GAS are classified by the *emm* protein on their surface, corresponding to the *emm* type. Certain *emm* types are linked to specific presentations [14,16]. WGS is recommended for comprehensive genetic characterization of GAS. There is often a strong association between specific *emm* types and GAS STs [17]. In our cases, the isolated strains were ST28 and ST433. ST28 has commonly been associated with *emm1*, a strain frequently seen in cases of invasive GAS infection [18]. An association of ST433 with a specific *emm* type is not as well documented. It is possible that more aggressive virulence factors present in ST28 compared with ST433 contributed to a worse clinical outcome for the first patient compared to the second, regardless of treatment. In considering the potential pathogenic mechanisms for the lysis of the lens zonules and sclera, the *emm1* strain frequently carries proteases, such as streptococcal pyrogenic exotoxin B (SpeB), a cysteine proteinase capable of degrading various host proteins, including components of the extracellular matrix. In addition, SpeB can activate matrix metalloproteinases, thereby facilitating lens zonule lysis [18]. Furthermore, synergistic effects from additional virulence factors, notably the cytolysins streptolysin O and streptolysin S, may contribute to the tissue destruction in these infections.

One limitation of our study is the lack of a positive culture from the eye in the second case, which may affect the robustness of our conclusions. This could be due to several factors, including prior antibiotic use or sampling error, as small volumes are a known limitation of eye samples [19]. Despite this limitation, the clinical presentation and response to treatment strongly suggest active GAS infection rather than an immune-mediated response only as aetiology.

In conclusion, our cases underscore the importance of early intravitreal antibiotic injection in managing GAS infections, even when involvement appears confined to the anterior segment. Spontaneous, atraumatic crystalline lens dislocation or subluxation, without vitreous involvement, should raise the index of suspicion for potential hyperacute ocular GAS, as this finding is atypical in more common aetiologies of endophthalmitis. Prompt intervention may prevent severe outcomes such as vision loss, phthisis and scleral perforation.

## Supplementary material

10.1099/acmi.0.000903.v4 Fig. S1.

## Abbreviations

AST: antimicrobial susceptibility testing
ED: emergency department
GAS: group A *Streptococcus*
IOP: intraocular pressure
IVIG: intravenous immunoglobulin
SBA: sheep blood agar
SpeB: streptococcal pyrogenic exotoxin B
ST: sequence type
WGS: whole-genome sequencing
