# Supplementary material for: Crystalline lens dislocation as a presenting sign of Streptococcus pyogenes invasive infections
Source: Access Microbiol. 2025 May 27;7(5):000903.v4. doi: 10.1099/acmi.0.000903.v4 (PMC12117008; doi:10.1099/acmi.0.000903.v4)
Supplement: Fig. S1. [file acmi-7-00903-s001.pdf]

**Supplemental Figure 1a: Case 1 Clinical Timeline**

| | | | | | | | | | | | | |
|---|---|---|---|---|---|---|---|---|---|---|---|---|
| **Timeline/Days** | -1 | Presentation to Wilmer | 1 | 2 | 3 | 4 | 5 | 6 | 7 | 8 | 9 | 10 |
| **Location** | Outpatient | ED | ICU | | ICU → Floor | Floor | | Outpatient | | | | |
| **Antibiotics** | | Ceftriaxone 2 g iv every 12 hours | | | | | | | | | | |
| | | Linezolid 600 mg iv every 12 hours | | | | | | | | | | |
| | | | | | | Penicillin G 4 million units iv every 4 hours | | | | | | |
| | | | | | | | Amoxicillin 500 mg po every 8 hours | | | | | |
| **Diagnostic procedures** | | Conjunctival culture+ | | | | | | | | | | |
| | | Blood culture | | | | | | | | | | |
| **Therapeutic procedures** | Laser Peripheral Iridotomy | | | | | | | | | | | |
| | | | | | Enucleation | | | | | | | |

+ Same-day results revealed abundant Gram-positive cocci arranged in chains, and light growth of Group A Streptococcus

**Supplemental Figure 1b: Case 2 Clinical Timeline**

| Timeline/Days | Presentation to Wilmer | 1 | 2 | 3 | 4 | 5 | 6 | 7 | 8 | 9 | 10 | 11 | 12 | 13 | 14 |
|---|---|---|---|---|---|---|---|---|---|---|---|---|---|---|---|
| **Location** | ED → ICU | ICU | | ICU → Floor | Floor | | | | | | | Outpatient | | | |
| **Systemic treatment** | Cefepime 2 g iv q8h | Ceftriaxone 2 g iv q12h | | | | | | | | | | | | | |
| | Vancomycin 1 g iv q12h | Clindamycin 600 iv mg q8h | Clindamycin → Linezolid 600 mg iv q12h | Linezolid 600 mg po q12h | | | | | | | | | | | |
| | Metronidazole 500 mg iv q8h | | | | | | | | | | | | | | |
| | Doxycycline 100 mg iv q12h | | | | | | | | | | | | | | |
| | IVIG | | | | | | | | | | | | | | |
| | | | | | Prednisone 40 mg/day | | | | | | | | | | |
| **Diagnostic procedures** | Blood cultures+ | | | | | | | | | | | | | | |
| | Skin swabs and cultures++ | | | | | | | | | | | | | | |
| **Therapeutic procedures** | | Intravitreal ceftazidime 2 mg + clindamycin 1 mg | | Intravitreal vancomycin 1 mg + ceftazidime 2 mg + dexamethasone 400 mcg | | | | | | | Intravitreal ceftazidime 2 mg + dexamethasone 400 mcg | | | | Pars plana lensectomy & vitrectomy |

+ Same-day results revealed abundant Gram-positive cocci arranged in chains. Culture was positive for Group A Streptococcus 10 hours after collection

++ Same-day results revealed insufficient material for Gram. Culture was positive for Group A Streptococcus 48 h after collection
